# Role of sex hormones and their receptors on gastric Nrf2 and neuronal nitric oxide synthase function in an experimental hyperglycemia model

**DOI:** 10.1186/s12876-020-01453-2

**Published:** 2020-09-23

**Authors:** Jeremy Sprouse, Chethan Sampath, Pandu R. Gangula

**Affiliations:** 1 School of Graduate Studies, Meharry Medical College, Nashville, TN 37208 USA; 2 Department of ODS & Research, School of Dentistry, Nashville, TN 37208 USA

**Keywords:** Gastroparesis, Sex hormones, Estrogen receptors, Drug therapy, nNOS, Nrf2, Nitrergic relaxation

## Abstract

**Background:**

Gastroparesis, a condition of abnormal gastric emptying, is most commonly observed in diabetic women. To date, the role of ovarian hormones and/or gastric hormone receptors on regulating nitrergic-mediated gastric motility remains inconclusive.

**Aim:**

The purpose of this study is to investigate whether sex hormones/their receptors can attenuate altered Nuclear factor (erythroid-derived 2)-like 2 (Nrf2), neuronal Nitric Oxide Synthase (nNOS) expression and nitrergic relaxation in gastric neuromuscular tissues exposed to in-vitro hyperglycemia (HG).

**Methods:**

Gastric neuromuscular sections from adult female C57BL/6 J mice were incubated in normoglycemic (NG, 5 mM) or hyperglycemic (30 mM or 50 mM) conditions in the presence or absence of selective estrogen receptor (ER) agonists (ERα /PPT or ERβ: DPN); or non-selective sex hormone receptor antagonists (ER/ICI 182,780, or progesterone receptor (PR)/ RU486) for 48 h. mRNA, protein expression and nitrergic relaxation of circular gastric neuromuscular strips were assessed.

**Results:**

Our findings in HG, compared to NG, demonstrate a significant reduction in ER, Nrf2, and nNOS expression in gastric specimens. In addition, in-vitro treatment with sex hormones and/or their agonists significantly (*p < 0.05) restored Nrf2/nNOSα expression and total nitrite production. Conversely, ER, but not PR, antagonist significantly reduced Nrf2/nNOSα expression and nitrergic relaxation.

**Conclusions:**

Our data suggest that ER’s can regulate nitrergic function by improving Nrf2/nNOS expression in experimental hyperglycemia.

## Background

Gastrointestinal (GI) dysfunction occurs in as many as 20–50% of patients with diabetes mellitus (T1/T2DM) [1]. Diabetic gastroparesis is a syndrome of delayed gastric emptying (GE) in the absence of mechanical obstruction of the upper stomach, antrum body, lower pyloric sphincter, and duodenum. Gastroparesis patients often experience symptoms including severe nausea, vomiting, and abdominal pain [2, 3]. Emerging evidence strongly indicate that women and female rodents are likely to experience more severe disease manifestations of gastroparesis compared to males [3, 4]. In fact, women during their reproductive ages, tend to be disproportionately affected by gastroparesis because their stomach motility is slower to begin with, likely due to elevated levels of sex steroid hormones and nitric oxide [5, 6]. Interestingly, women comprise nearly 80% of the patient population, but this predisposition for Gp remains unclear. Moreover, our laboratory has extensively reported nNOS expression and NO-mediated gut relaxation to be the predominant mechanism severely compromised in female rodent models of diabetic gastroparesis [5, 7, 8]. GI dysmotility, in particular, contributes to malnutrition in diabetic patients offering poor glycemic control and oral drug bioavailability among other concerns. Although options exist, current treatment models include dietary modifications, oral drug therapy (antibiotics, dopamine-2 (D2) receptor antagonists), and surgery [9, 10]. However, despite extensive research, drug therapy that can improve gastric emptying and decrease symptoms, without too many side effects are limited. Since the underlying mechanisms of gastric dysmotility are poorly understood, it is imperative to work with experimental hyperglycemic conditions to establish translational relevance.

Gastric motility is a highly coordinated activity of smooth muscle contraction and relaxation originating from the enteric nervous system within the stomach and intestine. Gastric function is largely regulated by an (1) excitatory (cholinergic) and inhibitory (nitric oxide (NO)/nitrergic) neurotransmitters working directly on smooth muscles or electrical signals that originate from the interstitial cells of Cajal (ICCs) [11]. Nitrergic signaling, the principal non-adrenergic, non-cholinergic (NANC) inhibitory mechanism in the gastrointestinal tract, plays a critical role in the control of gastric accommodation and pyloric relaxation. Several lines of evidence suggest that loss of Interstitial Cells of Cajal (ICC), neuronal nitric oxide synthase (nNOS) function, and elevated oxidative stress are hallmarks of diabetic gastroparesis [5, 11, 12]. nNOS produces nitric oxide (NO), a neuronal messenger with diverse functions throughout the body, and potent regulator of smooth muscle relaxation. nNOS is expressed as four splice variants: nNOS α, β, λ and μ [13]. Results from our in-vivo studies have demonstrated that nNOS-mediated gastric motility is greater in healthy female compared to male rodents. Furthermore, diabetes induction impaired nNOSα activity in female, but not in male in stomach and duodenum [7, 14]. Other studies have shown that supplementation of 17 β-estradiol (E_2_) increases nNOS expression and nitrergic-mediated gastric motility in ovariectomized rats [15, 16]. Furthermore, nNOS requires an essential cofactor, tetrahydrobiopterin (BH_4_) for enzyme activity. BH_4_ is produced via two distinct pathways. In de novo biosynthesis, guanosine triphosphate (GTP) is converted into BH_4_ by GTP cyclohydrolase I (GCH-1). BH_4_ is also produced through oxidation of dihydrobiopterin (BH_2_) by dihydrofolate reductase (DHFR), termed as the salvage pathway [17]. However, it is not known whether sex hormones and/or their receptors alter nNOS activity, nitrergic-mediated gastric function, or BH_4_ synthesis enzyme expression in an experimental hyperglycemia.

Estrogens mediate their biological actions through their respective nuclear (genomic) and cytoplasmic/membrane (non-genomic, rapid) receptors. Estrogen receptors (ERs) have two classical subtypes, ERα and ERβ, expressed from two distinct genes. Several studies demonstrated that both ERs are localized in nerve cells of the gut [18, 19]. E_2_ has multiple beneficial actions that include neuroprotection, maintaining glucose homeostasis, and activation of NO synthesis in vascular smooth muscle [20–23]. Although many studies aim to understand how E_2_ (non-selective ER agonist) can promote various cellular effects, selective activation of ERs may prove useful in novel treatment options for many diseased conditions. In addition, many studies highlight the fact that selective activation of ERα or ERβ may produce similar or opposing effects in different tissues [24, 25].

Interestingly, estrogens are known to be protective against oxidative stress in various cell types, yet the association between hormones and oxidative stress in gastroparesis has been elusive [20, 26]. Nuclear factor (erythroid-derived 2)-like 2 (Nrf2), an antioxidant-responsive transcription factor, has emerged as a potent target for alleviating clinical manifestations of diabetes mellitus (DM), in addition to its regulation of gastric nitrergic function [27–29]. Under oxidative stress, Nrf2 induces transcription of Phase II genes for detoxification and neutralization of reactive oxygen species (ROS) to protect against oxidative damage as manifested in diabetes mellitus. One of the primary protective downstream targets of Nrf2 is heme oxygenase 1 (HO-1), which has been shown to be protective to NO synthases in the vasculature [30, 31]. Our previous studies have shown that activation of Nrf2 can regulate antioxidant mechanisms, neuronal nitric oxide (nNOS)-mediated gastric function, and estrogen receptor expression in obesity-induced diabetic gastroparesis [27]. However, the contribution of distinct estrogen receptors on gastric Nrf2, Nrf2-Phase II detoxifying enzymes and nNOS function is unknown in experimental hyperglycemia.

Taken together, the objective of this study is to investigate whether selective activation of gastric estrogen receptors improves gastric Nrf2-nNOS expression in an in-vitro experimental hyperglycemic model. We hypothesize that estrogen and selective ER agonists regulate Nrf2/nNOS-mediated gastric motility and emptying in diabetic females.

## Methods

All experiments were approved by the Institutional Animal Care and Use Committee (IACUC) at Meharry Medical College (MMC), in accordance with recommendations of the National Institutes of Health (NIH) Guide for the Care and Use of Laboratory Animals. Adult female C57BL/6 J (*n* = 48, 12–15 weeks, 18.93 ± 0.272 g) mice were purchased from Jackson Laboratories (The Jackson Laboratory, Bar Harbor, ME). All animals were housed in the institutional animal care vivarium under standard conditions (4 mice/cage, 12 h light cycle) and allowed access to food and water ad libitum.

### Estrus cycle assessment

To exclude interference from endogenous sex hormones, all mice used in this study were selected during the diestrus stage of the reproductive cycle. The diestrus stage has been characterized by lower sex hormone (E_2_ + P_4_) levels [32, 33]. On the morning of the experimental day, vaginal smears were performed by flushing with ~ 50 ul of sterile phosphate buffered saline (PBS). The fluid was then carefully placed onto a glass slide and observed for predominant cell types present under a light microscope at 10x magnification. All mice used for in-vitro studies were healthy and only handled for estrous cycle determination prior to euthanization; therefore we report no adverse events.

### Experimental design and tissue culture experiments

On the day of experimentation, groups of healthy mice (*n* = 4/group, 11 total groups) were euthanized via CO_2_ asphyxiation. Full-thickness stomach and duodenum specimens were immediately isolated and placed in oxygenated Kreb’s physiological buffer (pH, 7.4). Gastric neuromuscular strips were randomized and incubated in various conditions for 48 h and grouped accordingly: (1) normoglycemic (NG; DMEM (5.5 mM glucose)), (2) mannitol (MAN; DMEM + 50 mM mannitol). Two concentrations of hyperglycemia were utilized, (3) 30 mM (DMEM (5.5 mM) + 24.5 mM D-glucose) and (4) 50 mM (DMEM (5.5 mM) + 44.5 mM D-glucose) glucose. In a separate set of experiments, gastric neuromuscular tissues were exposed to concentrations of (5) estradiol-17-β (E_2_), (6) Progesterone (P_4_), (7) 4,4′,4″-(4-Propyl-[1H]-pyrazole-1,3,5-triyl) trisphenol (PPT), or (8) diarylpropionitrile (DPN) in (9) high glucose (30 mM) respectively. All the compounds screened at different concentrations were chosen based on previously published in smooth muscle function [24, 34–36]. Similarly, the effects of gastric sex hormone receptors with (10) ICI 182, 720 (non-selective ER antagonist) and (11) RU-486 (progesterone receptor antagonists) 48 h were observed in NG conditions. At the end of each incubation, tissue specimens were quickly blotted dry, weighed and snap frozen in dry ice.

### Total nitrite concentration estimation

At the end of 48 h tissue incubation, all media samples were collected and stored at − 80 °C. Total nitrite was measured from cultured media via colorimetric assay based on manufacturer’s protocol (BioVision, Inc., Milpitas, CA, USA).

### Organ Bath studies

Electric field stimulation (EFS)-induced NANC relaxation was studied in circular gastric antrum neuromuscular strips, as previously described [8, 27]. The serosa layer was removed; however, the mucosal layers were intact for our studies. Circular gastric antrum neuromuscular strips were mounted between two L-shaped tissue hooks in 5 mL water jacketed organ baths containing Krebs buffer (pH 7.4) at 37 °C and continuously bubbled with 95% O_2_, 5% CO_2_ (DMT-USA, Inc., Ann Arbor, MI). Tension for each neuromuscular strip was monitored with an isometric force transducer and analyzed by a digital recording system. A passive tension equal to 2 g was applied to each strip during the 1 h equilibration period through incremental increases (0.5 g, four times, at 15 min intervals). Neuromuscular strips were incubated with atropine, phentolamine, and propranolol (10 μM each) for 30 min to block adrenergic and cholinergic responses. Strips were precontracted with serotonin (5-HT 100 μM) and were exposed to EFS (1 ms pulse) for 1 min duration to elicit NANC relaxation. The antrum neuromuscular strips were stimulated at 2 Hz and the resulting changes in response were measured for the baseline nitrergic relaxation. Then the neuromuscular strips were exposed to HG (30 mM) for 90 mins and were measured for nitrergic relaxation. To determine the effect of exogenous ovarian hormones or ER activators on nitrergic function, gastric antrum neuromuscular strips were pre-incubated in the presence of 30 mM HG simultaneously with E_2_, P_4_, PPT, or DPN for 90 mins. After incubation, the NANC-mediated relaxation was recorded.

In a separate set of experiments, the effect of ICI 182, 720 (non-selective ER antagonist) incubation on nitrergic relaxation in NG was measured. In each study, relaxation response elicited by low-frequency (2 Hz) stimulus under NANC conditions was predominantly nitrergic in origin. The NO dependence of NANC relaxations was confirmed by preincubation with N-Nitro-L-arginine methyl ester hydrochloride (L-NAME, 30 min; 100 μM, Sigma, St. Louis, MO). At the end of the experiment, each muscle strip was blotted dry with filter paper and weighed. Comparisons between the groups were performed by measuring the area under the curve (AUC) of the EFS-induced relaxation (AUCR) for 1 min and the baseline for 1 min (AUCB), according to the formula: (AUCR-AUCB)/weight of the tissue (mg) = AUC/mg tissue.

### RT-qPCR

Gastric antrum neuromuscular tissues were harvested from the treatment groups were snap frozen in liquid nitrogen. Total RNA was extracted using TriZol-Reagent (Thermo Fisher Scientific, Waltham, Ma) as described by the manufacturer’s protocol (Molecular Research Center, Thermo Fisher, Waltham, Ma). The iScript cDNA synthesis kit (Bio-Rad) was used to synthesize cDNA. One microliter of cDNA was used for each reaction, and the primers represented in Table 1 were used. RT-quantitative PCR (RT-qPCR) amplification was performed using the SYBR Green (Bio-Rad, Hercules, Ca) method. Cycling conditions were 95 °C for 3 min, followed by 45 cycles of 95 °C for 30 s and 55 °C for 1 min. Relative amounts of mRNA were normalized to β-actin and threshold cycle (CT) numbers were calculated (i.e., 2–ΔΔCT, the Ct method), according to the manufacturer’s instructions (Bio-Rad). All studies were performed in the MMC Molecular Core Laboratory.

**Table 1:** List of Primers used for quantitative RT-PCR assays

Gene	Forward	Reverse
ER⍺	5′- CCTCCCGCCTTCTACAGGT-3′	5′- CACACGGCACAGTAGCGAG-3′
ERβ	5′-CTGTGATGAACTACAGTGTTCCC-3’	5′-CACATTTGGGCTTGCAGTCTG-3’
nNOS⍺	5′-GGTGGAGATTAACATTGCTGTCCTA-3′	5′-TTCTCCATGTGTTTGATGAAGGACT-3′
Nrf2	5′-TCTCCTCGCTGGAAAAAGAA-3′	5′-TAAAGCACAGCCAGCACATT-3’
DHFR	5′-TCGACCATTGAACTGCATCGTCGCC-3’	5′-GGAATGGAGAACCAGGTTTTCCTACC-3’
GCH-1	5′- GAGCATCACCTTGTTCCATTTG − 3′	5′- GCCAAGTTTACTGAGACCAAGGA − 3′
β-Actin	5′-TGGAATCCTGTGGCATCCATGAAAC-3	5′-TAAAACGCAGCTCAGTAACAGTCCG-3′

### Western blot analysis

Full-thickness gastric neuromuscular specimens were homogenized, and protein were estimated in each of the lysates. Equal concentrations of protein lysates (40 μg) were separated on a 6 and 12% SDS polyacrylamide gel then transferred to nitrocellulose membrane. Each membrane were blocked with 5% dried non-fat milk for 1 h, then incubated with primary polyclonal antibody [ERα, (1:500), ERβ (1:500), DHFR (1:500), Nrf2 (1:1000), and HO-1 (1:1000) purchased from (Santa Cruz Biotechnology, Santa Cruz, Ca)] overnight, respectively. Expression of nNOSα was detected using rabbit polyclonal antibody at 1:1000 dilution (Abcam, Cambridge, Ma). The membranes were washed 3 times for 10 min each in 0.01% TBS-Tween, then incubated in horseradish peroxidase-conjugated secondary antibody (1:1000) for 1 h at room temperature. The blots were visualized with ECL Western Blotting Detection Reagent (GE Healthcare Bio-Sciences Corp., Piscataway, NJ) and ChemiDoc Touch Imaging system (BioRad, Hercules, CA). The reactive bands were analyzed quantitatively by optical densitometry. The blots were stripped and re-probed to measure protein expression. Membranes were washed three times in TBS buffer for 10 min. Membranes were then submerged in Restore Western Blot stripping buffer (Thermo Scientific, Rockford, Il) and gently rocked for 15 min. Blots were washed with TBS, three times for 10 min, before blocking with 5% dry milk in TBST buffer. Blots were re-probed with β-actin polyclonal antibodies (1:5000) (Sigma, St. Louis, MO) to enable normalization of signals between samples. Band intensities from Western blot images were analyzed using Image Lab Software (BioRad, Hercules, CA).

### Statistical analysis

Data were presented as the mean ± standard error (SE). For this study, we employed a method of sample size calculation based on the “resource equation” method, as previously detailed [37]. Statistical comparisons between groups were determined by the Student’s t-test or Tukey’s test after one-way Analysis of Variance (ANOVA), using the GraphPad Prism Version 5.0 (GraphPad Software, San Diego, CA). A *p*-value of less than 0.05 was considered statistically significant.

## Results

### Effect of estrogen receptor antagonist, ICI 182,780 and progesterone receptor antagonists, RU-486, on gastric nitrergic relaxation in normoglycemic conditions

**Fig. 1:**
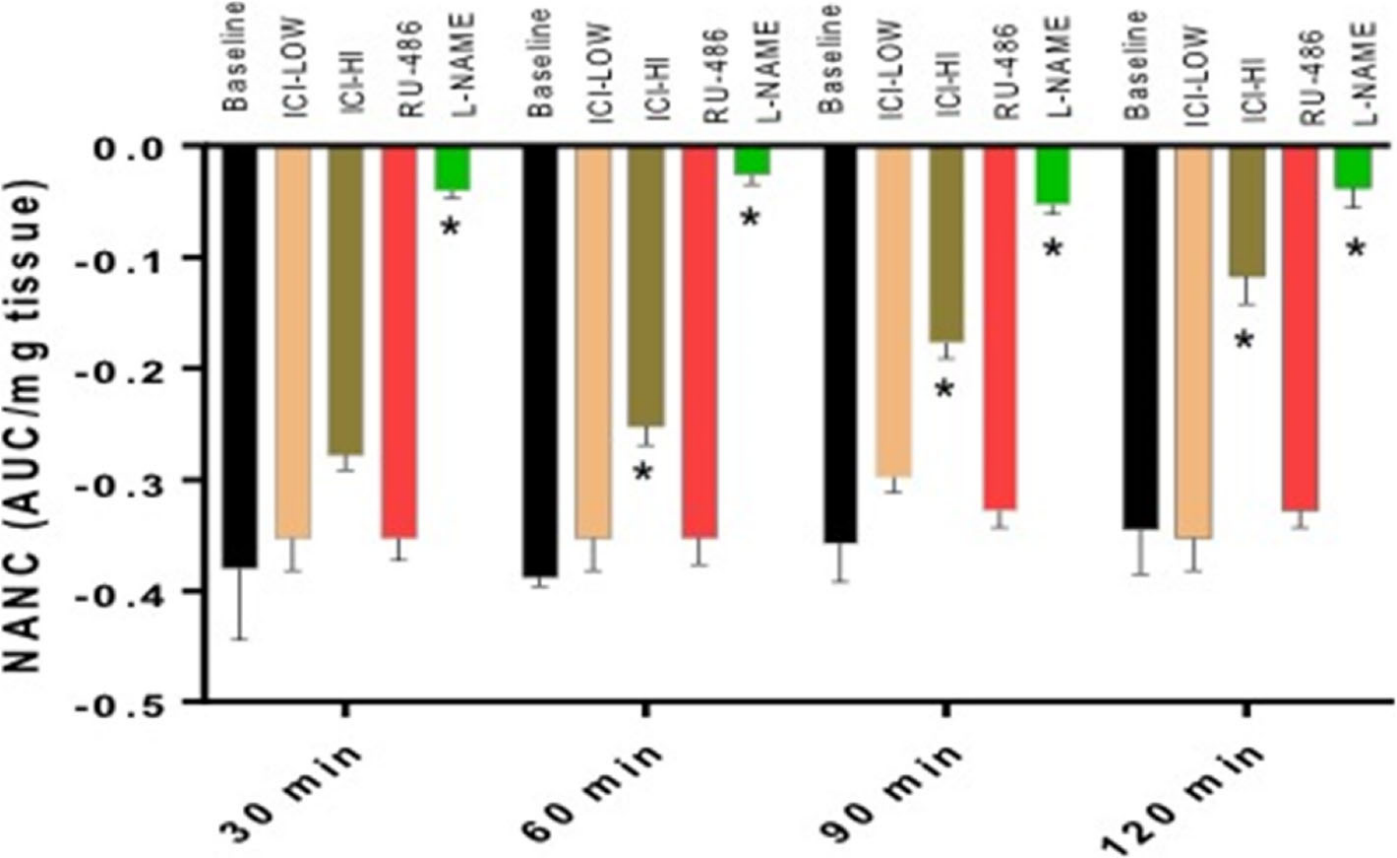
Effect of sex hormone receptor antagonist (ICI 182,780 and RU-486) on nitrergic relaxation during 2-h period. EFS (2 Hz) was used to elicit nNOS-mediated nitrergic relaxation from gastric antrum strips every 30 min during a 2-h period. Bar graphs showed changes in nitrergic relaxation with various treatments and expressed as area under the curve calculated per milligram tissue weight (AUC/mg tissue). Data were analyzed using one-way ANOVA by using GraphPad prism software. Data are means ± SEM (*n* = 4). * *p* < 0.05 compared with normal glycemia (NG) groups

Gastric neuromuscular specimens exposed to ICI 182,780 (1 nM, 10 nM) significantly (*p* < 0.05) reduced nitrergic relaxation. As shown in Fig. 1, this effect was measurable at 10 nM concentration as early as 60 mins, persisting to 120 mins (ICI 10 nM vs. baseline). RU-486 (100 μM), a common progesterone antagonist did not alter nitrergic relaxation in these tissues during the 2-h protocol.

### Effects of estrogen and progesterone receptor antagonist on gastric nNOS, Nrf2 and BH_4_ biosynthesis enzymes (GCH-1/DHFR) expression

**Fig. 2:**
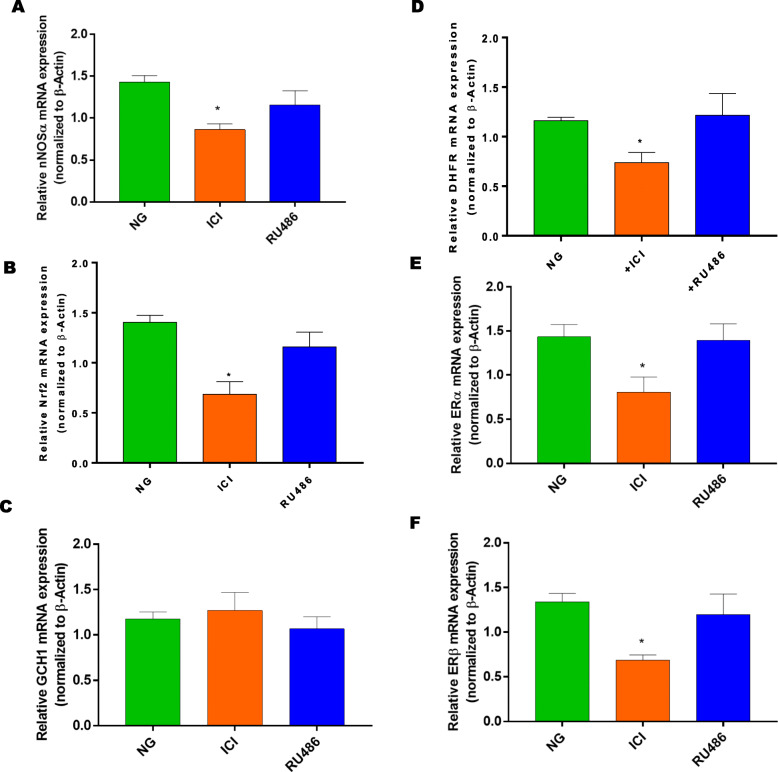
Effect of sex hormone receptor antagonist (ER; ICI 182,780 and PR; RU-486) on NOSα, Nrf2, ER and BH_4_ synthesis enzyme mRNA expression. Gastric neuromuscular tissues incubated with sex hormone receptor antagonists for 48 h (**a**) nNOSα, (**b**) Nrf2, (**c**) GCH-1, (**d**) DHFR, (**e**&**f**) ERα, ERβ mRNA expression were measured via RT-qPCR. Data were normalized with housekeeping gene (β-actin). Bar graphs showed a ratio of target gene or protein with β-actin. Data were analyzed using one-way ANOVA by using GraphPad prism software. Data are means ± SEM (*n* = 4). * *p* < 0.05 compared with normal glycemia (NG) groups

**Fig. 3:**
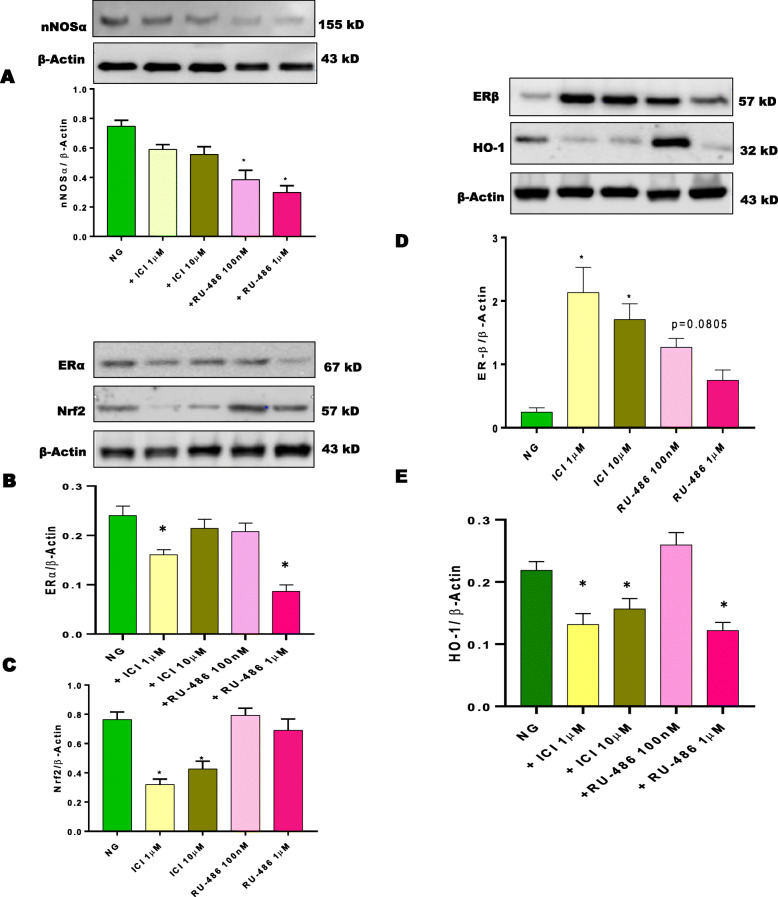
Effect of sex hormone receptor antagonist (ER; ICI 182,780 and PR; RU-486) Nrf2/nNOSα expression. Western blot analysis of relative protein expression of (**a**) nNOSα, (**b**) ERα, (**c**) Nrf2, (**d**) ERβ, (**e**) HO-1 are shown. Stripped blots were re-probed with β-actin. Data were normalized with housekeeping protein (β-actin). Bar graphs showed a ratio of target gene or protein with β-actin. Data were analyzed using one-way ANOVA by using GraphPad prism software. Data are means ± SEM (*n* = 4). * *p* < 0.05 compared with normal glycemia (NG) groups

To explore the role of sex hormone receptors on gastric Nrf2/nNOSα expression, gastric neuromuscular tissues were incubated in NG conditions for 48 h either with common ER antagonist, ICI 182,780 or PR antagonist, RU-486. Alterations in mRNA and protein expression were presented in Fig. 2. Non-selective blockade of gastric ERs with ICI 182,780 reduces gastric (Fig. 2a) nNOSα, (Fig. 2b) Nrf2, (Fig. 2c), DHFR and (Fig. 2e-f) estrogen receptor (α/β) mRNA expression. No measurable effect of RU-486, the progesterone receptor antagonist, was observed when compared to DMEM (NG) control group. In addition, no changes were observed with GCH-1 with either group. Similarly, protein expression of (Fig. 3a) nNOSα, (Fig. 3b) ERα, and HO-1 were significantly reduced in the presence of sex hormone antagonists, ICI 182,780 and RU-486. As shown in Fig. 3c, Nrf2, expression was reduced with ER antagonism. Interestingly, ERβ expression (Fig. 3d) was significantly (*p*< 0.05) increased in the presence of ICI 182,780 when compared to the control group. At lower concentrations of RU-486, no measurable change was observed in (Fig. 3e) HO-1 expression.

### Sex hormones and selective ER activation restored hyperglycemia-induced reduction of nitrergic relaxation in gastric antrum specimens

**Fig. 4:**
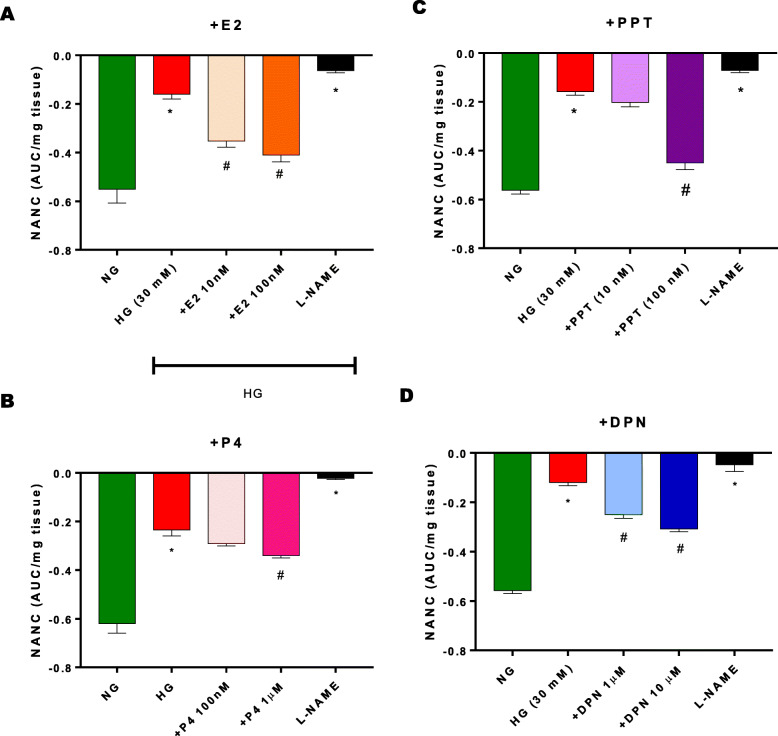
Effect of sex hormones and selective ER agonists (ERα; PPT, ERβ; DPN) on nNOS-mediated nitrergic relaxation in an in-vitro hyperglycemia model. (**a**-**d**) Neuromuscular strips were stimulated via EFS (2 Hz) after 90-min hyperglycemia incubation. Sex hormones (E_2_ or P_4_) or ER-selective agonists (PPT, DPN) were pre-incubated for 90 mins in hyperglycemia prior to second stimulation. L-NAME, nNOS blocker, was used to confirm nitrergic response. Bar graphs showed a ratio (AUC/mg tissue). Data were analyzed using one-way ANOVA by using GraphPad prism software. Data are means ± SEM (n = 4). * *p* < 0.05 compared with NG groups. # *p* < 0.05 compared with HG groups

Since ER antagonists reduced nitrergic relaxation in NG conditions, we next investigated whether E_2_ and/or ERs restore impaired (Fig. 4a-d) nitrergic relaxation in gastric antrum specimens exposed to in-vitro HG. Circular gastric antrum neuromuscular strips were mounted and incubated in hyperglycemia in the absence or presence of 17β-estradiol (E_2_) (Fig. 4a), progesterone (Fig. 4b), or selective ER agonists, PPT (Fig. 4c) or DPN (Fig. 4d). Nitrergic relaxation was significantly (*p* < 0.05) impaired in hyperglycemia. E_2_, at both concentrations (10 nM, 100 nM), restored nitrergic function. While activation of ERα with PPT effectively attenuated hyperglycemia-induced nitrergic dysfunction in neuromuscular strips at 100 nM concentration only (Fig. 4c), ERβ activation with DPN rescued nitrergic relaxation at both concentrations (1 μM, and 10 μM) (Fig 4d). Progesterone did not alter nitrergic relaxation in hyperglycemia. To confirm the relaxation response was nitrergic in origin, neuromuscular specimens incubated with L-NAME (nNOS blocker) display attenuation of gastric relaxation.

### Sex hormones and selective ER agonists restore Nrf2/ nNOSα expression and total nitrite production in hyperglycemic conditions

**Fig. 5:**
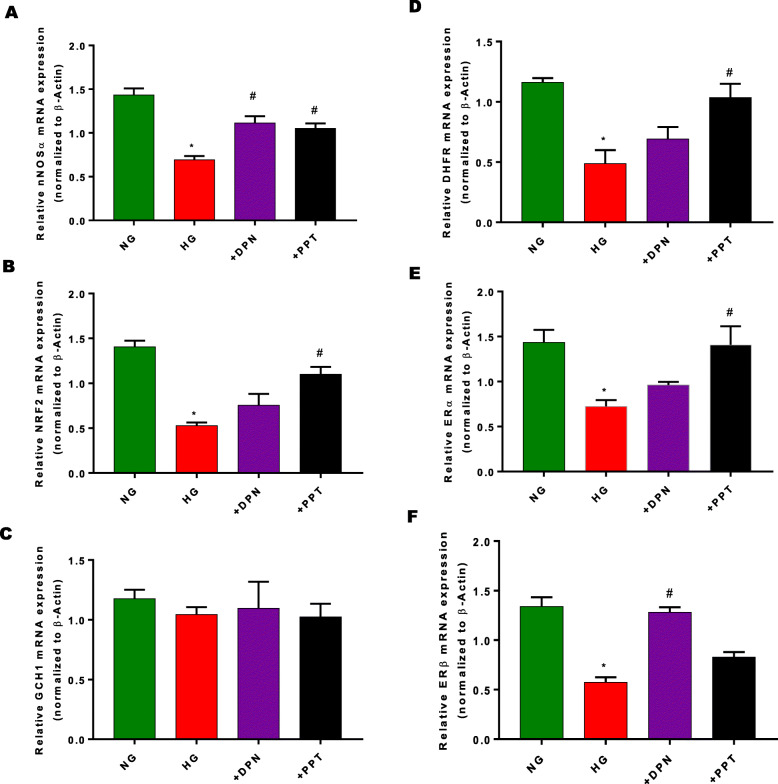
Effect of sex hormones and selective ER agonists (PPT, DPN) on Nrf2/nNOS, ERs, and BH_4_ synthesis enzyme mRNA expression in an in-vitro hyperglycemia model. (**a-f**) nNOS, Nrf2, ERs and BH4 synthesis enzyme mRNA expression. Gastric neuromuscular tissues incubated with selective ER-agonists for 48 h. (**a**) nNOSα, (**b**) Nrf2, (**c**) GCH-1, (**d**) DHFR, (**e**) ERα, and (**f**) ERβ mRNA expression were measured via RT-qPCR. Data were normalized with housekeeping gene (β-actin). Bar graphs showed a ratio of target gene or protein with β-actin. Data were analyzed using one-way ANOVA by using GraphPad prism software. Data are means ± SEM (n = 4). * *p*< 0.05 compared with normal glycemia (NG) groups

**Fig. 6:**
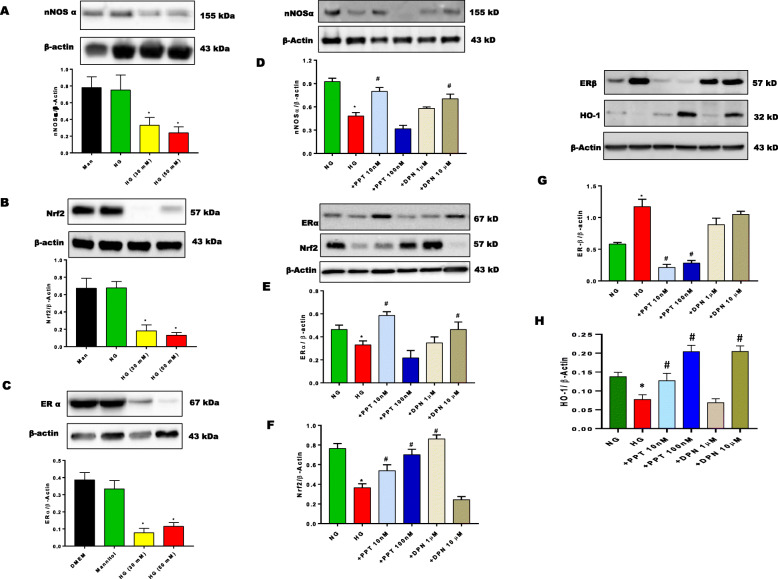
Effect of sex hormones and selective ER agonists (PPT, DPN) on Nrf2/nNOS, ERs, and HO-1 protein expression in 48 h in-vitro tissue culture. (**a**-**c**) The effect of 48 h in-vitro hyperglycemia was measured on nNOSα, Nrf2, ERα expression. In a separate set of experiments, the effect of PPT and DPN on (**d**-**h**) protein expression was assessed. Stripped blots were re-probed with β-actin. Data were normalized with housekeeping gene or protein (β-actin). Bar graphs showed a ratio of target gene or protein with β-actin. Data were analyzed using one-way ANOVA by using GraphPad prism software. Data are means ± SEM (n = 4). * *p*< 0.05 compared with NG groups. #  *p*< 0.05 compared with HG groups

Our data demonstrate a significant (*p* < 0.05) reduction in (Fig. 5a) nNOSα, (Fig. 5b) Nrf2, (Fig. 5c) GCH-1, (Fig. 5d) DHFR and (Fig. 5e-f) estrogen receptor (α/β) mRNA expression in hyperglycemic conditions. To assess the effect of hyperglycemia in gastric neuromuscular specimens, gastric neuromuscular specimens were incubated in the presence of high glucose (30 or 50 mM) for 48 h (Fig. 6a-c). The effects of elevated glucose levels on cellular osmolarity is reported [38, 39]. Thus, an isomer of glucose, mannitol was used as an osmotic control group in these studies [40]. Western blot analysis revealed no substantial change in the relative expression of (Fig. 6a) nNOS⍺, (Fig. 6b) Nrf2, or (Fig. 6c) ERα within the mannitol treated groups. Gastric tissues exposed to experimental hyperglycemia (30 mM or 50 mM), significantly (*p* < 0.05) reduced nNOS⍺, Nrf2, and ER⍺ levels. However, no differences were observed between hyperglycemic groups.

To assess whether sex hormones or selective ER activation altered Nrf2/nNOS protein expression, gastric neuromuscular tissues were incubated in hyperglycemia in the absence or presence of selective ER agonists, PPT or DPN. As seen in Fig. 6d-f, selective ER⍺/β activation (PPT/DPN) restored (Fig. 6d) nNOS⍺, (Fig. 6e) ER⍺, (Fig. 6f) Nrf2 protein expression in hyperglycemia. Moreover, (Fig. 6g) ERβ expression was significantly upregulated in hyperglycemia, but restored by PPT. HG altered HO-1 levels, changes were noted in the presence of PPT and DPN.

**Fig. 7:**
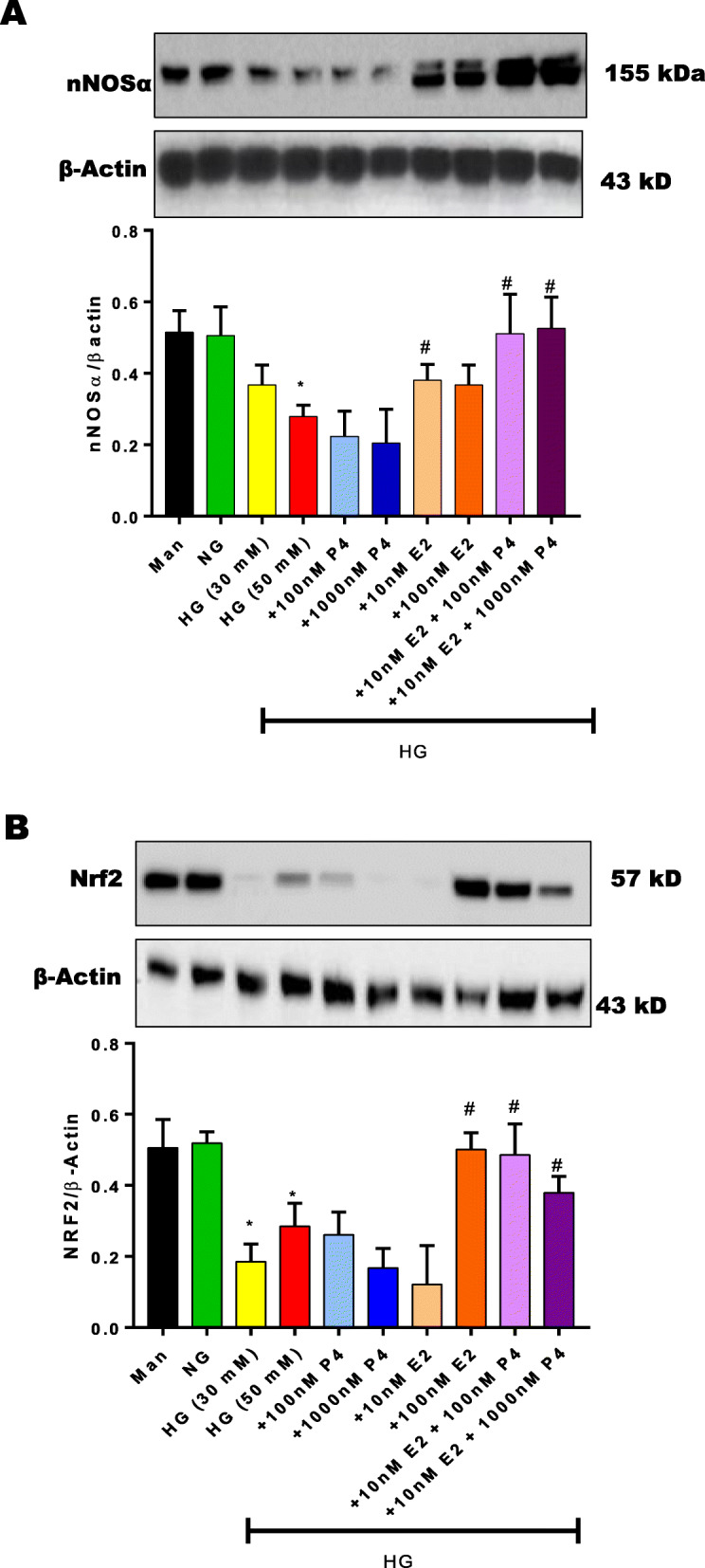
Effect of sex hormones on Nrf2/nNOS expression in duodenum specimens. (**a**) nNOS expression in hyperglycemia, (**b**) Nrf2 expression in 48 h in-vitro experiments. Stripped blots were re-probed with β-actin. Data were normalized with housekeeping gene or protein (β-actin). Bar graphs showed a ratio of target gene or protein with β-actin. Data were analyzed using one-way ANOVA by using GraphPad prism software. Data are means ± SEM (n = 4). * *p*< 0.05 compared with NG groups. # *p* < 0.05 compared with HG groups

**Fig. 8:**
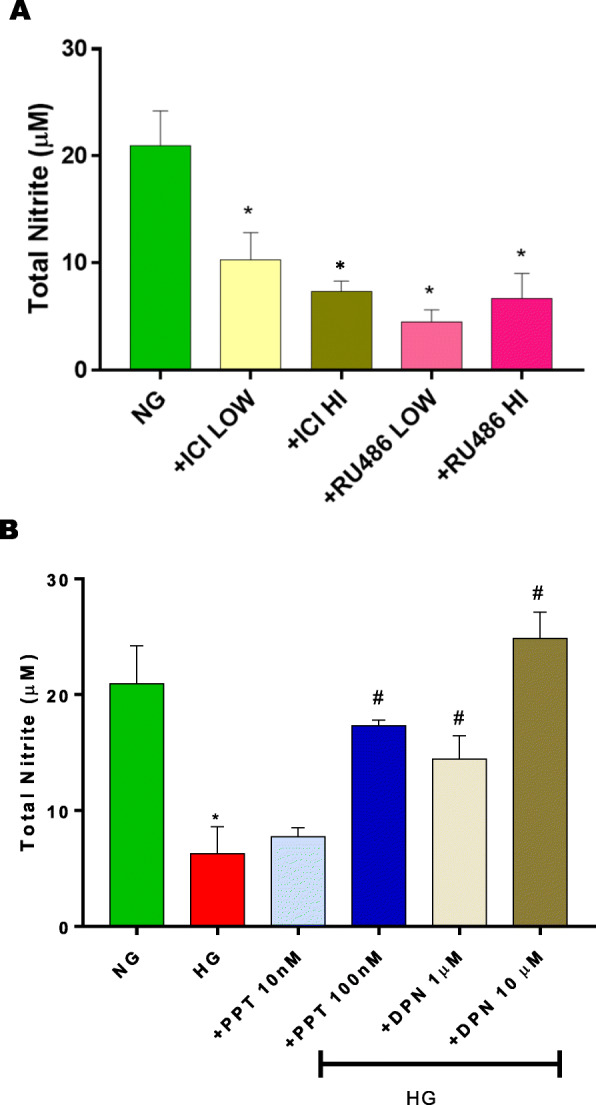
Effect of sex hormone antagonists (ICI 182,780 and selective ER agonists (PPT, DPN) on total nitrite production during 48 h incubation period from (**a**) normoglycemia (NG) or (**b**) hyperglycemia cultured media. Data were analyzed using one-way ANOVA by using GraphPad prism software. Data are mean ± SEM (n = 4). * *p*< 0.05 compared with NG groups. # *p* < 0.05 compared with HG groups

Since duodenal motility contributes to overall gastric motility and emptying function, we next investigated whether Nrf2/nNOSα expression is also affected in HG conditions. As shown in Fig. 7, in-vitro hyperglycemia significantly (p < 0.05) reduced (Fig. 7a) nNOS⍺ and (Fig. 7b) Nrf2 expression in duodenum specimens. As shown in Fig. 8a, sex hormone antagonists, ICI 182,780 and RU-486 decreased total nitrite production in-vitro. Similarly, total nitrite levels, a measure of NO production were significantly reduced in hyperglycemia. (Fig. 8b) PPT was effective at the higher concentration (100 nM) only. In contrast, DPN effectively attenuated reduced nitrite levels in hyperglycemic conditions at both concentrations (1 μM, and 10 μM).

## Discussion

Gender bias in gastric emptying is well-documented in both health and gastroparesis; however, the role of endogenous sex hormones in regulating gastric motility remains unclear. Women during their reproductive ages, tend to be disproportionately affected by gastroparesis because their stomach motility is slower to begin with, likely due to elevated levels of sex steroid hormones and nitric oxide. The pathogenesis of diabetes-associated motility disorders are multifactorial, though much can be attributed to abnormalities in nitric oxide/nNOS expression, enzyme activity and oxidative stress [41]. Our results show that in-vitro hyperglycemia reduces 1) gastric and duodenal Nrf2, nNOSα and ER alpha protein expression, 2) supplementation of estrogen and/or estrogen receptors restores Nrf2, nNOSα, total nitrite and nitrergic relaxation in hyperglycemic conditions. In addition, pre-incubation with ER antagonists inhibit Nrf2 and nNOSα protein expression and nitrergic relaxation. Collectively, the above studies suggest that estrogens regulate gastric motility via stimulating Nrf2/nNOSα signaling mechanism(s).

Estrogen receptor signaling is complex, but it is well understood to primarily mediate many of their biological effects via genomic regulation. Most of the actions of estrogens appear to be exerted via two estrogen receptor (ER) subtypes, denoted ERα and ERβ, intracellular proteins that are members of a large superfamily of proteins that function as ligand-activated transcription factors [22]. Although ER subtype surface membrane receptors exist, few studies have implicated these targets in the rapid vasodilator effects of E_2_ [23]. Moreover, several lines of evidence suggest that both ERα and ERβ proteins are expressed in enteric neurons within the nucleus and cytoplasm [19]. Co-expression of ERα and ERβ in enteric neurons indicates that estrogenic effects could also be mediated through neurogenic reflexes [16, 18]. Therefore, our study sought to understand the genomic changes associated with selective ER activation in gastric neuromuscular specimens.

To test whether sex hormone receptors contribute to gastric nitrergic function, we have first investigated the effects of non-selective antagonists: ICI 182, 780 (ER) and RU-486 (PR) in ex-vivo normoglycemic conditions. Our studies demonstrate that blockade of estrogen receptors, but not progesterone receptors, by antagonists showed a reduction in nitrergic relaxation in gastric neuromuscular strips; implicating ER signaling/effects. Estrogens have been shown to mediate both, rapid and genomic, effects. Genomic effects of steroid hormones have been shown to occur in as early as 2 h [42]. The results from our study revealed that mRNA and protein expression for Nrf2/nNOSα are altered by inhibition with ER or PR antagonists which clearly shows that total nitrite production is also altered in the presence of ICI and RU-486. The above data suggests that ERs could play a vital role in gastric motility function through regulating Nrf2/nNOSα expression and nitrergic relaxation.

Furthermore, several cofactors have been shown to be important for nNOSα activity, including BH_4_. The level of BH_4_ is tightly regulated by both de novo and salvage pathways. GCH-1 is a rate limiting enzyme and regulates BH_4_ levels via the de novo pathway, while DHFR reduces oxidized (inactive) BH_2_ and B to active BH_4_ via the salvage pathway. Previous studies from our laboratory suggest E_2_ deficiency reduced expression of GCH-1 and DHFR levels in female follitropin receptor knockout (FORKO) gastric neuromuscular tissue [8]. In addition, reduced levels of E_2_ may augment impairment of BH_4_-nNOSα function and elevate oxidative stress, thus promoting gastroparesis in women. Our studies show that inhibition of ER, but not PR, reduces DHFR, but not GCH-1 suggesting that ERs perhaps synthesizing BH_4_ via the salvage pathway.

Our current studies demonstrate that in-vitro exposure to HG significantly reduced gastric protein expression of both ERα and ERβ, Nrf2/nNOSα, total nitrite and nitrergic relaxation. Although the osmolarity of the culture medium was not assessed, the effects of elevated glucose levels on cellular osmolarity is well reported. Exposure to high glucose concentrations in vitro is often used to understand the cellular modifications occurring in diabetes [39]. Furthermore, recent studies have documented that hyperosmolarity, as occurring in diabetic hyperglycemia, may represent important regulatory elements influencing cell fate and viability, both in physiological and pathological conditions. These studies further report using glucose concentrations between 24 mM – 75 mM with incubation times up to 72 h are sufficient to mimic the diabetic oxidative stress response in different cellular types (human gingival fibroblasts and erythrocytes) [39, 43]. Furthermore, exposure to a prolonged severe hyperglycemic (> 30 mM) load is correlated with increased susceptibility to cellular damage and severe inhibitory effects on nNOS/Nrf2. However, short term incubation (< 48 h) display little effect on cell viability while maintaining HG insult. In our study, we examined the gastric neuromuscular response to increasing concentrations of hyperglycemia (DMEM (5.5 mM), 30 mM and 50 mM glucose) in the culture media for 48 h. (Fig. 6a-c). Although we observed no significant differences between nNOS, ER, and Nrf2 protein expression in 30 mM and 50 mM glucose media, the remaining HG experiments (incubation with hormones/ER agonists/antagonist and organ bath studies) employed a lower (30 mM) glucose concentration in the incubation media.

In addition, our studies show that nonselective (E_2_), and selective activation of ERα by PPT or ERβ by DPN restored gastric Nrf2/nNOSα expression, total nitrite and nitrergic relaxation in vitro exposed to HG. After 90 min incubation, we observe differences in the efficacy of the selective ER agonists at various doses; though each ER agonists significantly enhanced nitrergic relaxation in gastric neuromuscular specimens exposed to in-vitro hyperglycemia. This is in line with Al-Shboul et al., reporting that estrogen-induced relaxation was greater in female gastric smooth muscle cells (GSMC) compared with that in male [44]. Our current studies further asserted that ERα agonist, PPT and the ERβ agonist, DPN induced relaxation to a greater extent than PPT, although this result was not statistically significant. These differences may be due to variations in receptor subtype expression in GSMCs vs enteric neuronal cells. All of our data were generated from gastric full-thickness specimens that has several cellular subtypes including smooth muscle and enteric neuronal cells. Furthermore, as shown in Fig. 2, selective activation with PPT restored Nrf2, nNOSα, ERα, dihydrofolate reductase (DHFR) mRNA expression; selective ERβ activation (DPN) restored nNOS⍺, Nrf2, and ER⍺ mRNA expression. Collectively, the above studies suggest ER activators regulate nitrergic relaxation by restoring gastric Nrf2/BH_4_ synthesis in an experimental hyperglycemic condition.

Estrogen receptors (α/β) are encoded by separate genes and exhibit distinct tissue distributions [18, 23, 45–47]. ERα is well known to be majorly expressed in the uterus, liver, kidney and heart. Whereas, ERβ expression is primarily found in the ovary, lung, gastrointestinal tract, bladder, and central nervous systems. Much of the evidence describes spatially-specific actions in which ERs may possess converging or diverging pathways leading to cellular responses [27, 42]. It has been demonstrated in bone that ERβ can stimulate some of the same genes as does ERα, whereas ERβ almost always reduced the magnitude of gene stimulation by ERα when both receptors were present. It has also been reported through the use of subtype-selective ligands that ERβ modulation of ERα activity appears to be response specific. At experimental doses, PPT displays 410-fold selectivity for ERα over ERβ, whereas DPN displays a 70-fold selectivity for ERβ [34, 48]. However, Tamir et al. reported that oxidative stress, a well-known consequence of diabetes, differentially regulates the expression of ERα and ERβ in various cells [22]. Furthermore, although not studied in this context, numerous mRNA splice variants exist for both ERs in diseased and healthy tissues. These splice variants are speculated to potentially alter full-length (active) protein expression and activity in rodent studies, potentially facilitating compensatory signaling mechanism [49–51]. Our findings demonstrate that ERβ is increased in hyperglycemia. We anticipate that PPT activation of ERα may restore other mechanisms (i.e inflammation) to prevent the upregulation of ERβ in HG. Future studies are needed to further understand the complexities of sex hormone receptor signaling. Furthermore, the expression of ERs, DHFR, Nrf2, and HO-1 could be from mucosal, muscle and neuronal layers. We did not conduct the cellular localization studies of the target proteins in the current study. Of note, several studies have demonstrated that nNOS and ERs are primarily localized within enteric neuronal cells of the gut [16, 52]. Furthermore, regionally specific co-localizations of nNOS and ERα have also been reported, suggesting potential interaction in this system within the neurons. Reports indicate that enteric neurons innervate throughout the layers of the stomach [12, 18, 19]. Therefore, we speculate that despite of localization of these specific markers in various cell types (mucosal - > neurons), may interact with via autocrine and/or paracrine fashion, to thus regulate nNOS-mediated motility of the stomach.

Moreover, our previous studies demonstrate that loss of Nrf2 reduced nitrergic relaxation and delayed gastric emptying in female mice [27, 28]. In-vivo activation of Nrf2 has been shown to regulate gastric nNOSα function and ERs in a high-fat diet fed obesity Type II DM model [27]. Much of the work delineating the interactions between Nrf2 and ovarian hormone receptors is limited to breast cancer models [53, 54]. Estrogen (E_2_) increases Nrf2 activity in MCF7 breast cancer cells through activation of the PI3K/GSK3β non-genomic pathway [53]. Similarly, E_2_ is known to regulate, to a lesser extent, antioxidant response element (ARE)-responsive genes through Nrf2 and co-activators within the promoter region of these genes [53, 55, 56]. Our study sought to provide evidence for genomic changes in Nrf2 and nNOS in response to selective ER activation.

In addition, Nrf2 is widely known to bind to a host of Phase II detoxifying enzymes to rid the cell from oxidative stressors. Most importantly, heme oxygenase 1 (HO-1) is a protective marker controlled by Nrf2-ARE. In mouse models of diabetes, increased expression of antioxidants such as HO-1 protected ICC from oxidative stress and reversed diabetic gastroparesis [31]. Here, we provide evidence of a reduced expression of HO-1, that was restored by selective ER activators, suggesting ERα and β can increase HO-1 expression. These findings suggest co-activation with nuclear ERs can facilitate HO-1 gene expression.

Furthermore, motor abnormalities associated with gastroparesis syndrome may not be limited to the smooth muscle function. Diabetic gastroparesis comprises a decrease in fundic and antral motor activity, a reduction or a lack of the inter-digestive migrating motor complex, gastric dysrhythmias, and pylorospasms [13, 57]. As reported earlier, NO donors were ineffective in relieving gastroparesis symptoms in humans suggesting that stomach motility is not solely regulated by smooth muscle [58]. Numerous studies demonstrated that gut motility is regulated by enteric neuronal system including nNOS. Therefore, we suggest that although NO donors have an effect on relaxing smooth muscle in general, it may not relieve gastroparesis symptoms. In our study, hyperglycemia significantly reduced the expression of Nrf2 and nNOSα in gastric antrum and duodenum specimens. We further observed attenuation of Nrf2 and nNOSα protein expression with sex hormone (primarily E_2_) supplementation. Earlier studies in duodenum report the number of nNOS nerve cell bodies per ganglia was increased in type II DM rodent models of gastroparesis; however, the density index of nNOS varicosities was reduced [14, 59]. These studies further suggested that nitrergic neurons might be protected from hyperglycemia-related oxidative stress better in the duodenum. Similarly, the impairment of nNOS pathways in streptozotocin (STZ)-induced diabetic rats, the nitrergic myenteric neurons did not diminish in the duodenum, unlike the other gut segments [31]. In the diabetic duodenum, besides a decreasing number of nNOS neurons, the number of colocalized myenteric neurons did not alter significantly. Our studies so far align with a decreased expression of nNOS in the duodenum, however sex hormones may improve complications associated with this depletion of NO. It should be noted that Cellek, and others, suggest that nNOS expression is reversibly decreased (point of return) in the nitrergic axons while unaffected in the cell bodies in the early stages of diabetes in male rodents [60]. The same studies further suggest that nNOS neurons are reduced in the long-term diabetes (point of no return). Since hormones and their receptor agonists were able to restore nNOS protein expression, we suggest that our experimental HG model is related to the early stages, but not long-term diabetic conditions in which we would expect a loss of nNOS neurons.

## Conclusions

Taken together, these findings imply that direct regulation of multiple cellular molecules by estrogens may contribute to the modulation of gastric functions that have been recognized during hyperglycemic conditions. In the current study, we demonstrate the relevance of sex hormones and gastric estrogen receptors in mediating nitrergic relaxation and Nrf2/nNOS expression- both critical to gastrointestinal motility. Experiments are underway to investigate the in-vivo effects of hyperglycemia and sex hormones/receptor modulators on Nrf2- phase II antioxidant enzyme expression in stomach and duodenum. Linking symptoms to physiology and to cellular changes are important steps in determining the key targets for therapy design. A comprehensive identification of signaling pathways involving sex hormone receptors and Nrf2 activators on nitrergic function in diabetic rodent model may be beneficial targets to alleviate symptoms associated with diabetic gastroparesis in females. Drugs that can selectively modulate the activity of either ERα or ERβ in their interactions with target genes may also represent a promising frontier in gastric dysmotility coadjuvant therapy. Taken together, these findings provide insight into gender-related differences observed in gastric motility via experimental hyperglycemia.

## Data Availability

The datasets used and/or analyzed during the current study available from the corresponding author on reasonable request.

## Abbreviations

Nrf2: Nuclear factor (erythroid-derived 2)-like 2
nNOS: neuronal Nitric Oxide Synthase
HG: hyperglycemia
NG: normoglycemic
ER: estrogen receptor
PR: progesterone receptor
GI: Gastrointestinal
T1/T2DM: diabetes mellitus
GE: gastric emptying
D2: dopamine-2
NO: nitric oxide
ICCs: interstitial cells of Cajal
NANC: non-adrenergic, non-cholinergic
BH_4_: 17 β-estradiol (E_2_), tetrahydrobiopterin
GTP: guanosine triphosphate
GCH-1: GTP cyclohydrolase I
BH_2_: dihydrobiopterin
DHFR: dihydrofolate reductase
ROS: reactive oxygen species
IACUC: Institutional Animal Care and Use Committee
MMC: Meharry Medical College
NIH: National Institutes of Health
P_4_: progesterone
PBS: phosphate buffered saline
MAN: mannitol
PPT: 4,4′,4″-(4-Propyl-[1H]-pyrazole-1,3,5-triyl) trisphenol
DPN: diarylpropionitrile
DMEM: Dulbecco’s modified eagles media
EFS: Electric field stimulation
5-HT: serotonin
L-NAME: N-Nitro-L-arginine methyl ester hydrochloride
AUC: area under curve
RT-qPCR: Real-Time quantitative Polymerase Chain Reaction
CT: threshold cycle
ANOVA: Analysis of Variance
FORKO: follitropin receptor knockout
GSMC: gastric smooth muscle cells
ARE: antioxidant response element
HO-1: heme oxygenase 1
STZ: streptozotocin

## References

[1] Montgomery PA. Gastrointestinal complications of diabetes mellitus. J Pharm Care Pain Symptom Control. 1999;7:11–35. 10.1300/J088v07n02_03.

[2] Pratha VS (2004). Principles of gender-specific medicine.

[3] Kalra S, Sharma A, Priya G. Diabetic Gastroparesis. Diabetes Ther. 2018;9:1723–8. 10.1007/s13300-018-0475-4. 10.1007/s13300-018-0475-4 PMC6167284 30027528

[4] Kong M-F, Horowitz M. Gastric emptying in diabetes mellitus: relationship to blood-glucose control. Clin Geriatr Med. 1999;15:321–38. 10.1016/S0749-0690(18)30062-4. 10339636

[5] Gangula PRR, Sekhar KR, Mukhopadhyay S. Gender bias in gastroparesis: is nitric oxide the answer? Digestive Dis Sci. 2011. 10.1007/s10620-011-1735-6 PMC3170494 21559738

[6] Rao JN. Estrogens and Gastroparesis: a clinical relevance. Dig Dis Sci. 2013;58:1449–51. 10.1007/s10620-013-2683-0. 10.1007/s10620-013-2683-0 23625290

[7] Gangula PRR, Maner WL, Micci M-A, Garfield RE, Pasricha PJ. Diabetes induces sex-dependent changes in neuronal nitric oxide synthase dimerization and function in the rat gastric antrum. Am J Physiol Liver Physiol. 2007;292:G725–33. 10.1152/ajpgi.00406.2006. 10.1152/ajpgi.00406.2006 PMC2786258 17347455

[8] Ravella K, Al-Hendy A, Sharan C, Hale AB, Channon KM, Srinivasan S, et al. Chronic estrogen deficiency causes gastroparesis by altering neuronal nitric oxide synthase function. Dig Dis Sci. 2013;58:1507–15. 10.1007/s10620-013-2610-4. 10.1007/s10620-013-2610-4 PMC3691310 23504347

[9] Ma J, Rayner CK, Jones KL, Horowitz M. Diabetic Gastroparesis. Drugs. 2009;69:971–86. 10.2165/00003495-200969080-00003. 10.2165/00003495-200969080-00003 19496627

[10] Horowitz M, O’Donovan D, Jones KL, Feinle C, Rayner CK, Samsom M. Gastric emptying in diabetes: clinical significance and treatment. Diabet Med. 2002;19:177–94. 10.1046/j.1464-5491.2002.00658.x. 10.1046/j.1464-5491.2002.00658.x 11918620

[11] Bornstein JC, Costa M, Grider JR (2004). Enteric motor and interneuronal circuits controlling motility. Neurogastroenterology and Motility.

[12] Al-Shboul O (2013). The importance of interstitial cells of cajal in the gastrointestinal tract. Saudi J Gastroenterol.

[13] Adeghate E, Al-Ramadi B, Saleh AM, Vijayarasathy C, Ponery AS, Arafat K, et al. Increase in neuronal nitric oxide synthase content of the gastroduodenal tract of diabetic rats. Cell Mol Life Sci. 2003;60:1172–9. 10.1007/s00018-003-2298-2. 10.1007/s00018-003-2298-2 PMC11146055 12861383

[14] Surendran S, Kondapaka SB. Altered expression of neuronal nitric oxide synthase in the duodenum longitudinal muscle-myenteric plexus of obesity induced diabetes mouse: implications on enteric neurodegeneration. Biochem Biophys Res Commun. 2005;338:919–22. 10.1016/j.bbrc.2005.10.039. 10.1016/j.bbrc.2005.10.039 16256069

[15] Shah S, Nathan L, Singh R, Fu YS, Chaudhuri G. E 2 and not P 4 increases NO release from NANC nerves of the gastrointestinal tract: implications in pregnancy. Am J Physiol Integr Comp Physiol. 2001;280:R1546–54. 10.1152/ajpregu.2001.280.5.R1546. 10.1152/ajpregu.2001.280.5.R1546 11294780

[16] Molero L, García-Durán M, Diaz-Recasens J, Rico L, Casado S, López-Farré A. Expression of estrogen receptor subtypes and neuronal nitric oxide synthase in neutrophils from women and men: regulation by estrogen. Cardiovasc Res. 2002;56:43–51. 10.1016/S0008-6363(02)00505-9. 10.1016/s0008-6363(02)00505-9 12237165

[17] Crabtree MJ, Hale AB, Channon KM. Dihydrofolate reductase protects endothelial nitric oxide synthase from uncoupling in tetrahydrobiopterin deficiency. Free Radic Biol Med. 2011;50:1639–46. 10.1016/j.freeradbiomed.2011.03.010. 10.1016/j.freeradbiomed.2011.03.010 PMC3121954 21402147

[18] Campbell-Thompson M, Reyher KK, Wilkinson LB. Immunolocalization of estrogen receptor α and β in gastric epithelium and enteric neurons. J Endocrinol. 2001;171:65–73. 10.1677/joe.0.1710065. 10.1677/joe.0.1710065 11572791

[19] Liu JYH, Lin G, Fang M, Rudd JA. Localization of estrogen receptor ERα ERβ and GPR30 on myenteric neurons of the gastrointestinal tract and their role in motility. Gen Comp Endocrinol. 2019;272:63–75. 10.1016/j.ygcen.2018.11.016. 10.1016/j.ygcen.2018.11.016 30502347

[20] Numakawa T, Matsumoto T, Numakawa Y, Richards M, Yamawaki S, Kunugi H. Protective action of Neurotrophic factors and estrogen against oxidative stress-mediated Neurodegeneration. J Toxicol. 2011;2011:1–12. 10.1155/2011/405194. 10.1155/2011/405194 PMC3135156 21776259

[21] Pare G, Krust A, Karas RH, Dupont S, Aronovitz M, Chambon P, et al. Estrogen receptor-α mediates the protective effects of estrogen against vascular injury. Circ Res. 2002;90:1087–92. 10.1161/01.RES.0000021114.92282.FA. 10.1161/01.res.0000021114.92282.fa 12039798

[22] Tamir S, Izrael S, Vaya J. The effect of oxidative stress on ERα and ERβ expression. J Steroid Biochem Mol Biol. 2002;81:327–32. 10.1016/S0960-0760(02)00115-2. 10.1016/s0960-0760(02)00115-2 12361722

[23] Mendelsohn ME (2002). Genomic and nongenomic effects of estrogen in the vasculature. American Journal of Cardiology.

[24] Cignarella A, Bolego C, Pelosi V, Meda C, Krust A, Pinna C (2009). Distinct roles of estrogen receptor- and in the modulation of vascular inducible nitric-oxide synthase in diabetes. J Pharmacol Exp Ther.

[25] Katzenellenbogen BS, Choi I, Delage-Mourroux R, Ediger TR, Martini PGV, Montano M (2000). Molecular mechanisms of estrogen action: selective ligands and receptor pharmacology. Journal of Steroid Biochemistry and Molecular Biology.

[26] Almeida M, Martin-Millan M, Ambrogini E, Bradsher R, Han L, Chen XD, et al. Estrogens attenuate oxidative stress and the differentiation and apoptosis of osteoblasts by DNA-binding-independent actions of the ERα. J Bone Miner Res. 2009;:091012153414059–45. 10.1359/jbmr.091017. 10.1359/jbmr.091017 PMC3153331 19821774

[27] Sampath C, Sprouse JC, Freeman ML, Gangula PR. Activation of Nrf2 attenuates delayed gastric emptying in obesity induced diabetic (T2DM) female mice. Free Radic Biol Med. 2019;135:132–43. 10.1016/j.freeradbiomed.2019.02.029 PMC6738571 30831189

[28] Mukhopadhyay S, Sekhar KR, Hale AB, Channon KM, Farrugia G, Freeman ML, et al. Loss of NRF2 impairs gastric nitrergic stimulation and function. Free Radic Biol Med. 2011;51:619–25. 10.1016/j.freeradbiomed.2011.04.044. 10.1016/j.freeradbiomed.2011.04.044 PMC3129370 21605664

[29] Uruno A, Furusawa Y, Yagishita Y, Fukutomi T, Muramatsu H, Negishi T, et al. The Keap1-Nrf2 system prevents onset of diabetes mellitus. Mol Cell Biol. 2013;33:2996–3010. 10.1128/MCB.00225-13. 10.1128/MCB.00225-13 PMC3719683 23716596

[30] Ndisang JF. Role of Heme Oxygenase in inflammation, Insulin-Signalling, Diabetes and Obesity. Mediators Inflamm. 2010;2010:1–18. 10.1155/2010/359732. 10.1155/2010/359732 PMC2872759 20508722

[31] Chandrakumar L, Bagyánszki M, Szalai Z, Mezei D, Bódi N. Diabetes-related induction of the Heme Oxygenase system and enhanced Colocalization of Heme Oxygenase 1 and 2 with neuronal nitric oxide synthase in Myenteric neurons of different intestinal segments. Oxid Med Cell Longev. 2017;2017:1–13. 10.1155/2017/1890512. 10.1155/2017/1890512 PMC5610792 29081883

[32] Cora MC, Kooistra L, Travlos G. Vaginal Cytology of the Laboratory Rat and Mouse. Toxicol Pathol. 2015;43:776–93. 10.1177/0192623315570339. 10.1177/0192623315570339 PMC11504324 25739587

[33] Gonzalez G. Determining the stage of the estrous cycle in female mice by vaginal smear. Cold Spring Harb Protoc. 2016;2016:pdb.prot094474. 10.1101/pdb.prot094474. 10.1101/pdb.prot094474 27480723

[34] Reslan OM, Yin Z, Do Nascimento GRA, Khalil RA. Subtype-specific estrogen receptor-mediated vasodilator activity in the cephalic, thoracic, and abdominal vasculature of female rat. J Cardiovasc Pharmacol. 2013;62:26–40. 10.1097/FJC.0b013e31828bc88a. 10.1097/FJC.0b013e31828bc88a PMC3664271 23429596

[35] Watanabe T, Akishita M, Nakaoka T, Kozaki K, Miyahara Y, He H, et al. Estrogen receptor β mediates the inhibitory effect of estradiol on vascular smooth muscle cell proliferation. Cardiovasc Res. 2003;59:734–44. 10.1016/S0008-6363(03)00496-6. 10.1016/s0008-6363(03)00496-6 14499875

[36] Gingerich S, Krukoff TL. Activation of ERβ increases levels of phosphorylated nNOS and NO production through a Src/PI3K/Akt-dependent pathway in hypothalamic neurons. Neuropharmacology. 2008;55:878–85. 10.1016/j.neuropharm.2008.06.058. 10.1016/j.neuropharm.2008.06.058 18652836

[37] Charan J, Kantharia N (2013). How to calculate sample size in animal studies?. J Pharmacol Pharmacother.

[38] Duffy A, Liew A, O’Sullivan J, Avalos G, Samali A, O’Brien T. Distinct effects of high-glucose conditions on endothelial cells of macrovascular and microvascular origins. Endothel J Endothel Cell Res. 2006. 10.1080/10623320600659997 16885062

[39] Viskupicova J, Blaskovic D, Galiniak S, Soszyński M, Bartosz G, Horakova L, et al. Effect of high glucose concentrations on human erythrocytes in vitro. Redox Biol. 2015;5:381–7. 10.1016/j.redox.2015.06.011. 10.1016/j.redox.2015.06.011 PMC4506982 26141922

[40] Anitha M, Gondha C, Sutliff R, Parsadanian A, Mwangi S, Sitaraman SV, et al. GDNF rescues hyperglycemia-induced diabetic enteric neuropathy through activation of the PI3K/Akt pathway. J Clin Invest. 2006;116:344–56. 10.1172/JCI26295. 10.1172/JCI26295 PMC1359053 16453021

[41] Mahavadi S, Sriwai W, Manion O, Grider JR, Murthy KS. Diabetes-induced oxidative stress mediates upregulation of RhoA/Rho kinase pathway and hypercontractility of gastric smooth muscle. PLoS One. 2017;12:e0178574. 10.1371/journal.pone.0178574. 10.1371/journal.pone.0178574 PMC5497948 28678840

[42] Marino M, Galluzzo P, Ascenzi P. Estrogen Signaling Multiple Pathways to Impact Gene Transcription. Curr Genomics. 2006;7:497–508. 10.2174/138920206779315737. 10.2174/138920206779315737 PMC2269003 18369406

[43] Buranasin P, Mizutani K, Iwasaki K, Pawaputanon Na Mahasarakham C, Kido D, Takeda K, et al. High glucose-induced oxidative stress impairs proliferation and migration of human gingival fibroblasts. PLoS One. 2018;13:e0201855. 10.1371/journal.pone.0201855. 10.1371/journal.pone.0201855 PMC6084939 30092096

[44] Al-Shboul OA, Nazzal MS, Mustafa AG, Al-Dwairi AN, Alqudah MA, Omar AA, et al. Estrogen relaxes gastric muscle cells via a nitric oxide- and cyclic guanosine monophosphate-dependent mechanism: a sex-associated differential effect. Exp Ther Med. 2018;16:1685–92. 10.3892/etm.2018.6406. 10.3892/etm.2018.6406 PMC6122185 30186388

[45] Barros RPA, Machado UF, Gustafsson JÅ. Estrogen receptors: new players in diabetes mellitus. Trends Mol Med. 2006;12:425–31. 10.1016/j.molmed.2006.07.004. 10.1016/j.molmed.2006.07.004 16890492

[46] Barros RPA, Gustafsson JÅ. Estrogen receptors and the metabolic network. Cell Metabolism. 2011;14:289–99. 10.1016/j.cmet.2011.08.005. 10.1016/j.cmet.2011.08.005 21907136

[47] Kuiper GGJM, Carlsson B, Grandien K, Enmark E, Häggblad J, Nilsson S, et al. Comparison of the ligand binding specificity and transcript tissue distribution of estrogen receptors and α and β. Endocrinology. 1997;138:863–70. 10.1210/endo.138.3.4979. 10.1210/endo.138.3.4979 9048584

[48] Song X, Pan ZZ (2012). Estrogen receptor-beta agonist diarylpropionitrile counteracts the estrogenic activity of estrogen receptor-alpha agonist propylpyrazole-triol in the mammary gland of ovariectomized Sprague Dawley rats. J Steroid Biochem Mol Biol.

[49] Herynk MH, Fuqua SAW. Estrogen receptor mutations in human disease. Endocrine Rev. 2004;25:869–98. 10.1210/er.2003-0010. 10.1210/er.2003-0010 15583021

[50] Heldring N, Pike A, Andersson S, Matthews J, Cheng G, Hartman J, et al. Estrogen receptors: how do they signal and what are their targets. Physiol Rev. 2007;87:905–31. 10.1152/physrev.00026.2006. 10.1152/physrev.00026.2006 17615392

[51] Pelekanou V, Kampa M, Kiagiadaki F, Deli A, Theodoropoulos P, Agrogiannis G, et al. Estrogen anti-inflammatory activity on human monocytes is mediated through cross-talk between estrogen receptor ERα36 and GPR30/GPER1. J Leukoc Biol. 2016;99:333–47. 10.1189/jlb.3A0914-430RR. 10.1189/jlb.3A0914-430RR 26394816

[52] Scordalakes EM, Shetty SJ, Rissman EF. Roles of estrogen receptor α and androgen receptor in the regulation of neuronal nitric oxide synthase. J Comp Neurol. 2002;453:336–44. 10.1002/cne.10413. 10.1002/cne.10413 12389206

[53] Wu J, Williams D, Walter GA, Thompson WE, Sidell N. Estrogen increases Nrf2 activity through activation of the PI3K pathway in MCF-7 breast cancer cells. Exp Cell Res. 2014;328:351–60. 10.1016/j.yexcr.2014.08.030. 10.1016/j.yexcr.2014.08.030 25172557

[54] Lo R, Matthews J. The aryl hydrocarbon receptor and estrogen receptor alpha differentially modulate nuclear factor erythroid-2-related factor 2 transactivation in MCF-7 breast cancer cells. Toxicol Appl Pharmacol. 2013;270:139–48. 10.1016/j.taap.2013.03.029. 10.1016/j.taap.2013.03.029 23583297

[55] Ansell PJ, Lo SC, Newton LG, Espinosa-Nicholas C, Zhang DD, Liu JH, et al. Repression of cancer protective genes by 17β-estradiol: ligand-dependent interaction between human Nrf2 and estrogen receptor α. Mol Cell Endocrinol. 2005;243:27–34. 10.1016/j.mce.2005.08.002. 10.1016/j.mce.2005.08.002 16198475

[56] Lee JM, Anderson PC, Padgitt JK, Hanson JM, Waters CM, Johnson JA. Nrf2, not the estrogen receptor, mediates catechol estrogen-induced activation of the antioxidant responsive element. Biochim Biophys Acta - Gene Struct Expr. 2003;1629:92–101. 10.1016/j.bbaexp.2003.08.006. 10.1016/j.bbaexp.2003.08.006 14522084

[57] Tack J, Van den Houte K, Carbone F. Gastroduodenal motility disorders. Curr Opin Gastroenterol. 2018;34:428–35. 10.1097/MOG.0000000000000473. 10.1097/MOG.0000000000000473 30199408

[58] Kashyap P, Farrugia G. Diabetic gastroparesis: what we have learned and had to unlearn in the past 5 years: Figure 1. Gut. 2010;59:1716–26. 10.1136/gut.2009.199703. 10.1136/gut.2009.199703 PMC2996823 20871131

[59] Stenkamp-Strahm CM, Nyavor YEA, Kappmeyer AJ, Horton S, Gericke M, Balemba OB. Prolonged high fat diet ingestion, obesity, and type 2 diabetes symptoms correlate with phenotypic plasticity in myenteric neurons and nerve damage in the mouse duodenum. Cell Tissue Res. 2015;361:411–26.10.1007/s00441-015-2132-9. 10.1007/s00441-015-2132-9 PMC4530039 25722087

[60] Cellek S. Point of NO return for Nitrergic nerves in diabetes: a new insight into diabetic complications. Curr Pharm Des. 2004;10:3683–95. 10.2174/1381612043382792. 10.2174/1381612043382792 15579064

